# Sacubitril–valsartan *versus* enalapril for the treatment of acute decompensated heart failure in Chinese settings: A cost-effectiveness analysis

**DOI:** 10.3389/fphar.2023.925375

**Published:** 2023-03-02

**Authors:** Tianyang Hu, Yiting Liu, Yake Lou

**Affiliations:** ^1^ Precision Medicine Center, The Second Affiliated Hospital of Chongqing Medical University, Chongqing, China; ^2^ Department of Critical Care Medicine, The Second Affiliated Hospital of Chongqing Medical University, Chongqing, China; ^3^ Department of Cardiology, The Second Affiliated Hospital of Chongqing Medical University, Chongqing, China

**Keywords:** sacubitril–valsartan, cost-effectiveness, acute decompensated heart failure, ADHF, heart failure

## Abstract

**Background:** The episode of acute decompensated heart failure (ADHF) is the main cause of hospitalization for heart failure (HF). Sacubitril–valsartan has been proven to be effective in reducing the risks of hospitalization for HF in ADHF. When to initiate sacubitril–valsartan in ADHF to make it the most cost-effective in China remains unclear.

**Methods:** A lifetime Markov model with a 1-month cycle length was developed to evaluate the cost-effectiveness of early or late initiation of sacubitril–valsartan *versus* enalapril in ADHF. Early initiation of sacubitril–valsartan meant that it was initiated after stabilization from ADHF, and late initiation of sacubitril–valsartan meant that it was initiated after stabilization from HF, which includes no hospitalization for at least three consecutive months. The primary outcome was the incremental cost-effectiveness ratio (ICER), expressed as the ratio of incremental cost to incremental effectiveness. The secondary outcomes were total costs and total effectiveness. Three times of *per capita* GDP of China in 2021 was set as the willingness-to-pay threshold. One-way sensitivity analysis and probabilistic sensitivity analysis were employed to test the robustness of the results.

**Results:** The early initiation of sacubitril–valsartan treatment resulted in an ICER of 3,662.4 USD per quality-adjusted life year, lower than the willingness-to-pay threshold, and the late initiation of sacubitril–valsartan treatment gained an ICER of 4,444.4 USD/QALY, still lower than the willingness-to-pay threshold. One-way sensitivity analysis showed that our results were robust, and probabilistic sensitivity analysis suggested that early initiation of sacubitril–valsartan in ADHF was cost-effective under a 97.4% circumstance.

**Conclusion:** Early initiation of sacubitril–valsartan after stabilization of ADHF is highly cost-effective compared with the use of enalapril; late initiation of sacubitril–valsartan after stabilization of HF is still cost-effective but not as cost-effective as early initiation of sacubitril–valsartan in ADHF. For Chinese ADHF patients, the time to initiate sacubitril–valsartan should be when the patient is stabilized from ADHF rather than when stabilized from HF, from the perspective of economic evaluation.

## Introduction

Heart failure (HF) is a terminal manifestation of many heart diseases. It is estimated that about 38 million patients suffer from HF worldwide (Braunwald, 2015). The incidence of HF increases dramatically with age. For the population aged over 40 years old, the incidence is about 1%–2%, but it increases to 10% in those over 70 years old (McDonagh et al., 2021). The reason for hospitalization for HF is mainly due to the episode of acute decompensated heart failure (ADHF) (Zhang et al., 2017). ADHF may cause serious consequences, including deterioration of heart function, repeated hospitalizations, and death (Cook et al., 2014). About 4.1% of ADHF patients with HF die during hospitalization (Zhang et al., 2017; Ma et al., 2021), and 20% of patients will be subsequently readmitted to a hospital 1 month after hospitalization for ADHF (Reddy and Borlaug, 2019; Lan et al., 2021). In China, there are about 8.9 million patients suffering from HF, and annually, 2.58 million Chinese patients die of HF (Lan et al., 2021).

Sacubitril–valsartan, as an angiotensin–neprilysin inhibition agent, has been proven to be superior to enalapril in reducing the risks of cardiovascular death and hospitalization for HF in HF patients with reduced ejection fraction (HFrEF) (McMurray et al., 2014). In HFrEF patients with ADHF, sacubitril–valsartan has also been demonstrated to reduce the risk of hospitalization for HF, but it has not proved to reduce mortality within 2 months after hospitalization (Velazquez et al., 2019). Although sacubitril–valsartan is superior to enalapril in HFrEF treatment, whether sacubitril–valsartan should be added into the standard treatment remains controversial (Liu et al., 2021) because some studies showed that sacubitril–valsartan is not cost-effective in their settings. In a study conducted in America, investigators found that adding sacubitril–valsartan into the standard treatment resulted in an incremental cost-effectiveness ratio of 143,891 USD per quality-adjusted life year, which is higher than the willingness-to-pay threshold, indicating that sacubitril–valsartan is not cost-effective in HFrEF (Zueger et al., 2018), but a study performed in Chinese settings suggested that sacubitril–valsartan is cost-effective in HFrEF (Wu et al., 2020). For the cost-effectiveness of sacubitril–valsartan in ADHF patients, there are still different conclusions. A study conducted in Australia proved that adding sacubitril–valsartan into standard treatment is not cost-effective in ADHF (Perera et al., 2021). However, Krittayaphong’s study found that sacubitril–valsartan is cost-effective in Thailand (Krittayaphong and Permsuwan, 2021). There is still no study performing the economic evaluation of sacubitril–valsartan in Chinese ADHF patients.

Cost-effectiveness analysis is a way of balancing the costs and benefits of new and traditional therapies. A new therapy is often associated with higher costs but better effectiveness. Cost-effectiveness analysis could answer the question of whether a new therapy is worth it or not. Considering the huge economic burden of HF in China, it is necessary for us to perform an economic evaluation to investigate the cost-effectiveness of sacubitril–valsartan *versus* enalapril in ADHF.

## Methods

The present study was reported in accordance with the Consolidated Health Economic Evaluation Reporting Standards 2022 (CHEERS 2022) Statement (Husereau et al., 2022).

### Population

The target population of the present study was a hypothesis cohort in China with similar baseline characteristics to those in the PIONEER-HF study (Velazquez et al., 2019). In the PIONEER-HF study, the patients had a median age of 62 years old, with an interquartile range of 51–72 years, a left ventricular ejection fraction (EF) of 40% or less, and an N-terminal pro–B-type natriuretic peptide (NT-proBNP) concentration of 1,600 pg per milliliter or more, or a B-type natriuretic peptide (BNP) concentration of 400 pg per milliliter or more, and had received a primary diagnosis of ADHF, including signs and symptoms of the fluid overload. The baseline characteristics of PIONEER-HF are shown in Table 1 and details of inclusion and exclusion criteria are shown in Table 1 in the supplementary materials.

**TABLE 1:** Baseline characteristics of patients included in the PIONEER-HF study.

Variable	Value
Age	62 (51–72)
Female sex (%)	28
Body mass index (kg/m^2^)	30.3 (25.8–37.1)
Previous heart failure (%)	65.4
Previous use of medication (%)	
ACE inhibitor or ARB	47.9
Beta blocker	59.6
MRA	10
Loop diuretic	57
Hydralazine	7.2
Nitrate	9.5
Digoxin	8.6
NYHA class (%)	
I	1
II	25.2
III	62.7
IV	8.6
Not assessed	2.5
Systolic blood pressure	118 (109–133)
Left ventricular ejection fraction (%)	24.5 (18–30)
NT-proBNP at randomization (pg/ml)	2710 (1,363–5403)
Medical history (%)	
Myocardial infarction	7
Atrial fibrillation	35.4
ICD only	19.8
CRTD	8.7
Comorbidities (%)	
Hypertension	85.5
Previous stroke	9.9
Diabetes mellitus	19.1
Hyperlipidemia	37.1

Abbreviations: ACE, angiotensin-converting enzyme; ARB, angiotensin receptor blocker; MRA, mineralocorticoid receptor antagonist.

**FIGURE 1:**
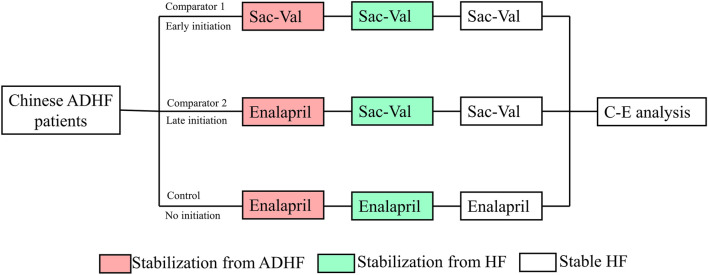
Abbreviation: ADHF, acute decompensated heart failure; Sac–val, sacubitril–valsartan; C-E. cost-effectiveness; HF, heart failure. Chinese ADHF patients were randomly allocated to receive early initiation of sacubitril–valsartan, late initiation of sacubitril–valsartan, or no initiation. After a lifetime simulation, the cost-effectiveness analysis was performed.

There were two comparators and one control; of the two comparators, one had an early initiation of sacubitril–valsartan, in which the sacubitril–valsartan treatment was initiated after stabilization from ADHF (comparator 1), defined by the maintenance of a systolic blood pressure of at least 100 mm Hg for the preceding 6 h, with no increase in the dose of intravenous diuretics, no use of intravenous vasodilators during the preceding 6 h, and no use of intravenous inotropes during the preceding 24 h. The other comparator had a late initiation of sacubitril–valsartan treatment, in which the treatment was initiated after stabilization from HF, defined as not being hospitalized for at least three consecutive months (comparator 2). The control was given enalapril from the start, and the same treatment was continued after discharge from hospitalization. All the subsequent sensitivity analyses were based on comparator 1. The research process is shown in Figure 1.

### Model construction

**FIGURE 2:**
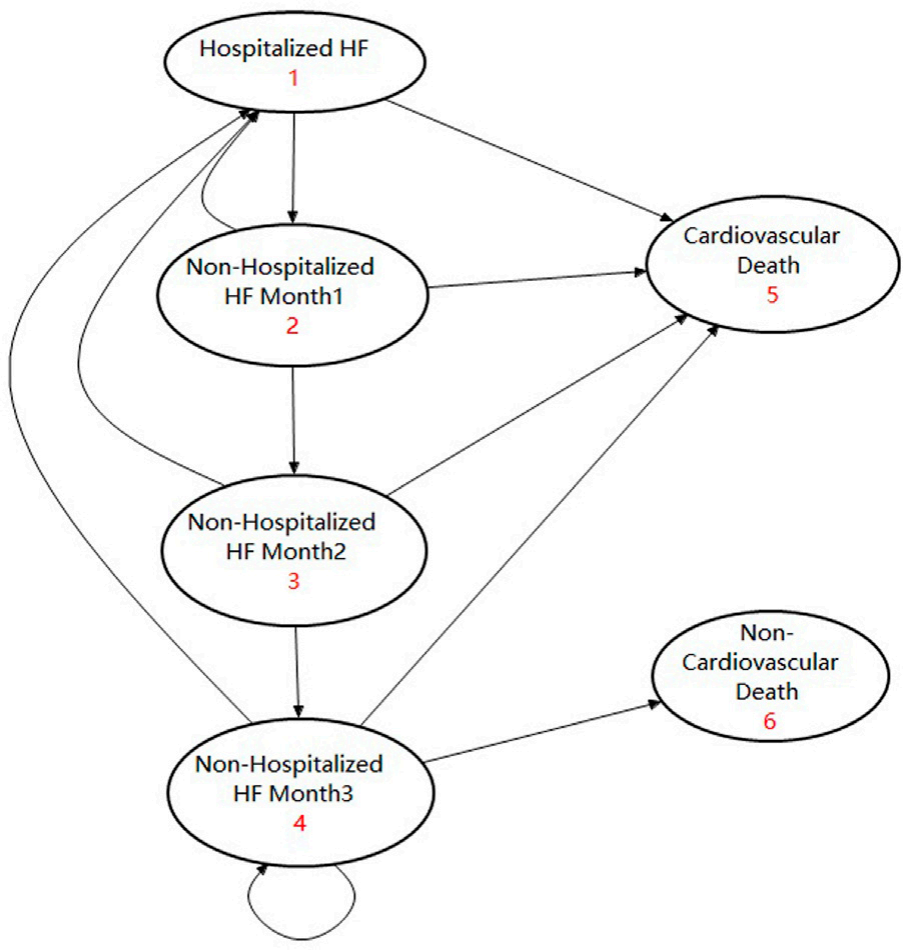
Abbreviation: HF, heart failure. Diagram of the Markov model using the state transition.

A lifetime horizon Markov model with a 1-month cycle was developed to evaluate the cost-effectiveness of early or late initiation of sacubitril–valsartan *versus* enalapril in ADHF. Considering the fact that the median age of patients in the PIONEER-HF study was 62 years old and that studies showed that the mean age of Chinese HFrEF patients was 60 years old, we took 60 years old as the starting age of the Markov simulation (Zhang et al., 2017; Velazquez et al., 2019). In the Markov model, hospitalized ADHF patients could receive sacubitril–valsartan 200 mg (97 mg of sacubitril plus 103 mg of valsartan) twice a day or enalapril 10 mg twice a day plus standard treatment, and the same treatment would continue after their discharge until the lifetime horizon (Velazquez et al., 2019). In our simulation, considering that readmission and cardiovascular death tended to occur within 3 months after discharge, we assumed that patients who had not been hospitalized for three consecutive months were in a stable state (Greene et al., 2015). Patients who had been hospitalized within 3 months had a higher incidence of rehospitalization and cardiovascular death, and a death occurring within 2 months after discharge was considered cardiovascular death. Patients stabilized for at least three consecutive months had a lower incidence of death and hospitalization, and they may experience cardiovascular death or non-cardiovascular death. Based on this assumption, there were four transition states and two absorbed states in our model, which were “Hospitalized HF,” “Non-hospitalized HF month 1,” “Non-hospitalized HF month 2,” “Non-hospitalized HF month 3,” “Cardiovascular death,” and “Non-cardiovascular death”. Patients who entered this model would begin with the “Hospitalized HF” state, and those who did not experience cardiovascular death would enter the transition state of “Non-hospitalized HF month 1.” Patients in “Non-hospitalized HF month 1” may experience cardiovascular death or readmission. Patients who did not experience such events would enter “Non-hospitalized HF month 2.” Patients in “Non-hospitalized HF month 2” may still experience cardiovascular death or readmission, and those who did not experience such events for at least three consecutive months would enter “Non-hospitalized HF month 3”. Patients in “Non-hospitalized HF month 3” were considered stable, and they may experience cardiovascular death, non-cardiovascular death, or hospitalization. Those who did not experience such events would continue the cycle in “Non-hospitalized HF month 3”. The model was validated by other studies, and the corresponding state transition diagram can be seen in Figure 2 (Gaziano et al., 2016; Krittayaphong and Permsuwan, 2021).

The study was performed from a Chinese healthcare system perspective. Only direct costs (drugs and hospitalization costs) were accounted for in our model. All the costs were converted to the price in 2021 in China, according to the consumer price indexes of healthcare (CPI). The CPIs in 2015–2021 were 1.027, 1.038, 1.06, 1.043, 1.024, 1.018, and 1.004, respectively. Future costs, life year (LY), and quality-adjusted life-years (QALYs) were discounted at a rate of 0.03 per year, which was the geometric mean value of the aforementioned figures, but non-monetary outcomes (hospitalization incidence, readmission incidence, and mortality rate) were not discounted. The discount rate ranged from 0 to 0.06 in the one-way sensitivity analysis.

### Parameter input

All input parameters are listed in Table 2.

**TABLE 2:** Input parameters of the Markov model.

Parameter	Base case	Range low	Range high	SD	Distribution	Source
Transition probability for cardiovascular death						
Sac–valsartan (≤2 months)^a^	0.0114	0.0044	0.0185	0.0035	β	Velazquez et al. (2019)
Enalapril (≤2 months)	0.0172	0.0086	0.0258	0.0044	β	Velazquez et al. (2019)
Sac–valsartan (≥3 months)^b^	0.0053	0.0048	0.0057	0.0002	β	McMurray et al. (2014)
Enalapril (≥3 months)	0.0066	0.0061	0.0071	0.0003	β	McMurray et al. (2014)
Transition probability for non-cardiovascular death						
60–64 years	0.0004	—	—	—	—	Ma et al. (2022)
65–69 years	0.0007	—	—	—	—	Ma et al. (2022)
70–74 years	0.001	—	—	—	—	Ma et al. (2022)
75–79 years	0.0017	—	—	—	—	Ma et al. (2022)
80–84 years	0.0026	—	—	—	—	Ma et al. (2022)
≥85 years	0.0054	—	—	—	—	Ma et al. (2022)
Transition probability for hospitalization						
Sac–valsartan (≤2 months)	0.04060	0.0275	0.0539	0.0067	β	Velazquez et al. (2019)
Enalapril (≤2 months)	0.0717	0.0545	0.0893	0.0089	β	Velazquez et al. (2019)
Sac–valsartan (≥3 months)^c^	0.0256	0.0228	0.0285	0.0014	β	McMurray et al. (2014)
Enalapril (≥3 months)^d^	0.0339	0.0306	0.0373	0.0017	β	McMurray et al. (2014)
Costs (**USD**/month)						
Sac–valsartan^e^	50.5	25.2	180.7	18	γ	Local institution
Enalapril + standard^f^	37.9	18.9	75.7	3.8	γ	Huang et al. (2017)
Hospitalization^g^	2361.5	1,180.7	4,722.9	236	γ	Huang et al. (2017)
Utilities						
Sac–valsartan (per month)^h^	0.0698	0.0628	0.0768	0.0036	β	Krittayaphong and Permsuwan (2021)
Enalapril (per month)	0.0691	0.0622	0.076	0.0035	β	Krittayaphong and Permsuwan (2021)
Hospitalization	-0.1	-0.08	-0.13	0.0128	β	Krittayaphong and Permsuwan (2021)
Discount rate^i^	0.03	0	0.06	—	—	—

Abbreviations: Sac–valsartan, sacubitril–valsartan; SD, standard deviation.

^a^ 1-month rate is 0.0115 = -ln (1–10/440)/2, and 1-month probability is 0.0114 = 1-exp (-0.0115).

^b^ 1-month rate is 0.0053 = -ln (1–558/4,187)/27, and 1-month probability is 0.0053 = 1-exp (-0.0053).

^d^ 1-month rate is 0.0345 = -ln (1–392/1,157)/12, and 1-month probability is 0.0339 = 1-exp (-0.0345).

^c^ 1-month rate is 0.026 = -ln (1–392*0.79/1,157)/12, and 1-month probability is 0.0256 = 1-exp (-0.0259589416,564,517).

^e^ 1-month sac–valsartan cost is 50.5 USD, 38 CNY/7 tablets * twice a day* 30 days/6.4515 (CNY/USD).

^f^ 1-month enalapril + standard cost is 37.9 USD = 28,974 CNY/year*0.082/12*1.027*1.038 *1.06 *1.043*1.024*1.018*1.004/6.4515 (CNY/USD).

^g^ Cost for one hospitalization event is 2361.5 USD = 12,351 CNY*1.027*1.038* 1.06* 1.043*1.024*1.018* 1.004/6.4515 (CNY/USD).

^h^ 1-month utility in sac–valsartan is 0.0698 = 0.838/12, and 1-month utility in enalapril is 0.0691 = 0.829/12.

^i^ Discount rate is 0.03 = power (1.027*1.038*1.06*1.043*1.024*1.018*1.004,1/7)-1.

### Transition probability input

For those who were in the transition states of “Hospitalized HF,” “Non-hospitalized HF month 1,” and “Non-hospitalized HF month 2,” the transition probabilities were calculated based on the PIONEER-HF study (comparison of sacubitril/valsartan versus enalapril on the effect on NT-proBNP in patients stabilized from an acute heart failure episode), which reported the cardiovascular mortalities and rehospitalization incidence for hospitalized ADHF patients. The 2-month cardiovascular mortality rate in sacubitril–valsartan was 0.023 = 10/440, and the 1-month cardiovascular mortality rate was 0.0115 = -ln (1–10/440)/2; then, the transition probability for 1-month cardiovascular death in sacubitril–valsartan was 0.0114 = 1-exp (-0.0115). Using the same formula, we calculated that the transition probability for 1-month cardiovascular death in enalapril was 0.0172, and the corresponding 1-month transition probabilities for rehospitalization in sacubitril–valsartan and enalapril were 0.0406 and 0.0717, respectively. Those who entered “Non-hospitalized HF month 3” were regarded stable, and the transition probabilities for cardiovascular death were derived from the PARADIGM-HF study (efficacy and safety of LCZ696 compared to enalapril on morbidity and mortality of patients with chronic heart failure). Using the aforementioned formula, the transition probabilities obtained for 1-month cardiovascular death in sacubitril–valsartan and enalapril were 0.0053 and 0.0066, respectively. The transition probability for hospitalization of patients in enalapril was obtained from a Chinese study investigating the economic burden of HF in China; the 1-year hospitalization rate was 0.3388 = 392/1,157, and the 1-month transition probability was 0.0339. For transition probability in sacubitril–valsartan, we used the hazard ratio (HR) and hospitalization rate in enalapril to calculate the rehospitalization rate in sacubitril–valsartan, and we found that the 1-month transition probability in sacubitril–valsartan was 0.0256.

The transition probability for non-cardiovascular death was accessed from the China Health Statistical Yearbook 2021 and was age-dependent (Ma et al., 2022). It was calculated using the total mortality rate minus the cardiovascular mortality rate as there is no non-cardiovascular mortality reported in the Yearbook. The yearly mortality was converted to a 1-month mortality rate by dividing it by 12. The 1-month non-cardiovascular mortality rates for those aged 60–64, 65–69, 70–74, 75–79, 80–84, and ≥85 were 0.0004, 0.0007, 0.0010, 0.0017, 0.0026, and 0.0054. Both cohorts adopted the same non-cardiovascular mortality rate.

### Cost input

The cost of sacubitril–valsartan was as per the collective purchasing price of the Chinese government, which was 38 Chinese yuan (CNY)/7 tablets (200 mg/tablets) (equal to 5.9 USD). Based on taking 200 mg twice a day, the monthly cost for sacubitril–valsartan was 325.7 CNY (equal to 50.5 USD). The lower interval of the cost of sac–valsartan was obtained, assuming that the cost of sacubitril–valsartan could reduce to 50% of its current price. For the upper interval, we adopted the price before sacubitril–valsartan was included in the collective purchasing list, namely 1,165.7 Chinese yuan/month (180.7 USD). The cost for enalapril plus standard treatment was obtained from Huang et al. (2017). The cost for enalapril plus standard treatment was 198 CNY (equal to 29.1 USD) per month in 2014, and it was 244.2 CNY (equal to 37.9 USD) per month in 2021, when taking the CPI into consideration. The cost for hospitalization is also derived from Huang et al. (2017); using the same method, we can conclude that the cost for hospitalization was 15,235 CNY (2361.5 USD) per event in 2021. To enable the reader an easy understanding of the cost-effectiveness of sacubitril–valsartan *versus* enalapril in Chinese ADHF patients, all costs were converted from CNY to USD, at a ratio of 6.4515, which was the average value of the exchange rate in 2021.

The adverse events in the PIONEER-HF study incurred low treatment costs, and it was not included in the analysis.

### Utility input

According to a published study, the utilities in sacubitril–valsartan and enalapril were 0.838 and 0.829, respectively. Every hospitalization event would result in a reduction of 0.1 in utility (-0.1/time).

### Outcomes

The primary outcome of the present study was the incremental cost-effectiveness ratio (ICER), expressed as the ratio of incremental cost to incremental effectiveness. Secondary outcomes are total costs and total effectiveness (life-years and quality-adjusted life-years (QALYs)). According to the recommendation of the China Guidelines for Pharmacoeconomic Evaluations (Hu et al., 2020), the willingness-to-pay (WTP) threshold was three times the current *per capita* GDP in China, which was 242,928 CNY = 80,976 CNY*3 (equal to 37,654.5 USD) (Wang et al., 2021). If the ICER calculated was lower than that threshold, it would be thought to be cost-effective; otherwise, it would not be cost-effective.

### Sensitivity analysis

One-way sensitivity and probabilistic sensitivity analyses were employed to validate the impacts of these parameters on outcomes and the robustness of our results. In the one-way sensitivity analysis, the parameters fluctuated in their 95% confidence interval (CI) or given interval, and a tornado diagram was drawn to display our results. In the probabilistic sensitivity analysis, 10,000 times of Monte Carlo simulations based on probabilistic sensitivity sampling were performed, and the results were illustrated in cost-effectiveness acceptability curves and scatter plots.

### Statistical analysis

All the statistical analyses were performed using TreeAge Pro 2011 software (Williamstown, MA. United States) and EXCEL software (Redmond, Washington, United States), and a half-cycle correction was applied in the model to prevent overestimation of the costs and effectiveness.

## Results

### Base case analysis

After a simulation of the lifetime horizon, the early initiation of sacubitril–valsartan treatment resulted in a higher cost than enalapril treatment but gained a higher QALY and life year, which incurred an ICER of 3,662.4 USD/QALY. The costs of sacubitril–valsartan and enalapril were 17,515.2 and 12,189.7 USD, respectively, and the incremental cost was 5325.4 USD. The QALYs in both groups were 7.28 and 5.82, respectively. For life years, sacubitril–valsartan still got higher life years than enalapril, which were 9.12 and 7.51 life years, respectively (Table 3).

**TABLE 3:** Base case analysis of sacubitril–valsartan *versus* enalapril for treatment of acute decompensated heart failure.

Intervention	Total cost (USD)	Total effectiveness (QALY)	Total effectiveness (LY)	Incremental cost (USD)	Incremental effectiveness (QALY)	ICER (USD/QALY)
Enalapril	12,189.7	5.82	7.51	-	-	-
HF hospitalization	8,920.9	-0.4	0	-	-	-
Stable state	3,268.9	6.22	7.51	-	-	-
Sac–val (early initiation)	17,515.2	7.28	9.12	5325.4	1.45	3,662.4
HF hospitalization	8,140.3	-0.36	0	-780.6	0.04	-
Stable state	9,374.9	7.64	9.12	6,106	1.42	-
Sac–val (late initiation)	16,483.6	6.79	8.55	4,293.9	0.97	4,444.4
HF hospitalization	8,085.7	-0.37	0	-835.2	0.03	-
Stable state	8,397.9	7.16	8.55	5129	0.94	-

Abbreviations: QALY, quality-adjusted life-year; LY, life year; ICER, incremental cost-effectiveness ratio; HF, heart failure; Sac–val, sacubitril–valsartan.

The late initiation of sacubitril–valsartan treatment still gained higher costs and higher QALY than enalapril treatment. The costs were 16,483.6 USD and 12,189.7 USD, respectively, and the corresponding effectiveness were 6.79 and 5.82 QALY, thus resulting in an ICER of 4,444.4 USD/QALY.

### One-way sensitivity analysis

**FIGURE 3:**
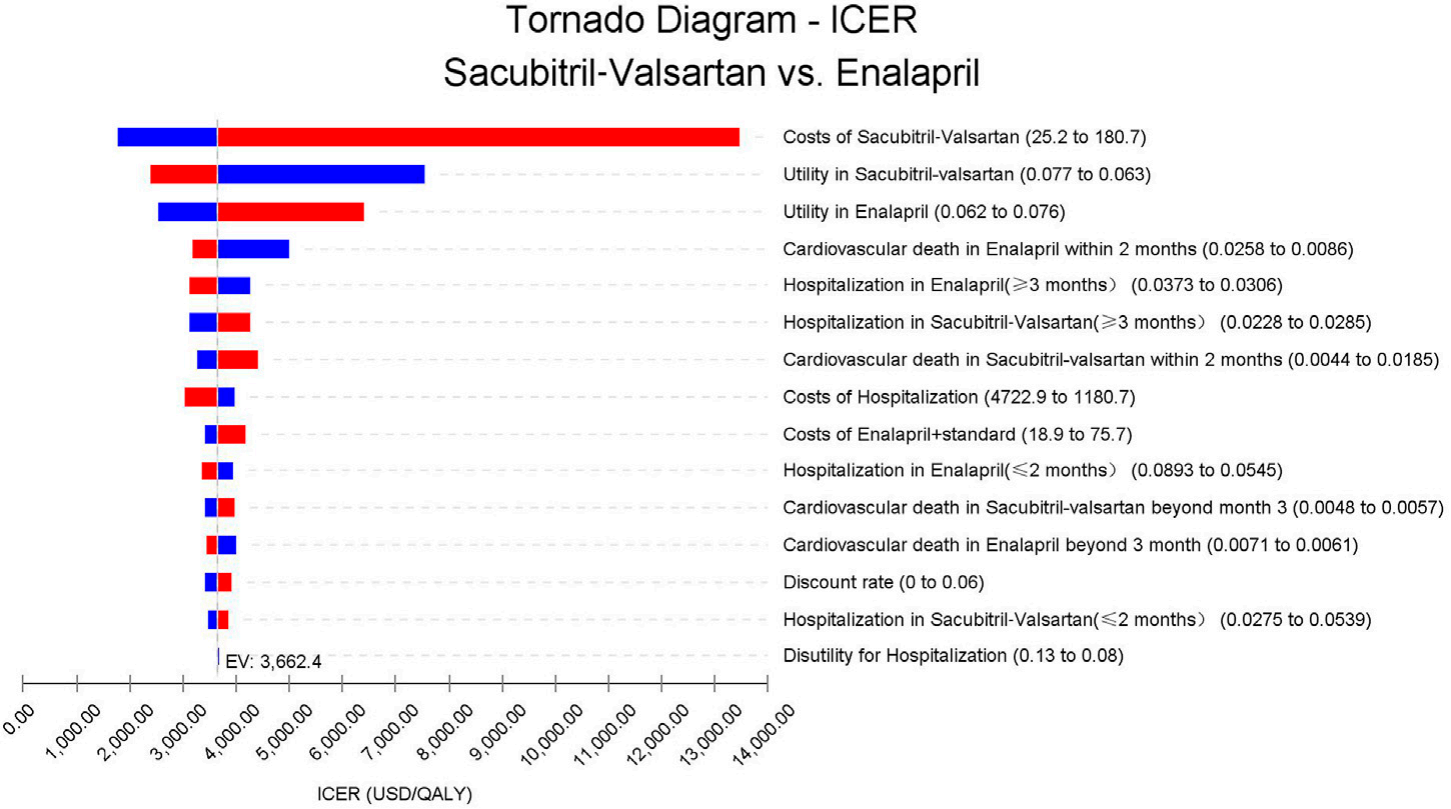
Abbreviation: ICER, incremental cost-effectiveness ratio. Tornado diagram based on the one-way sensitivity analysis. Costs of sacubitril–valsartan impact the most on the ICER fluctuation; other input parameters have little impact on ICERs.

As could be seen in Figure 3, the cost of sacubitril–valsartan had the largest impact on the ICER. When costs of sacubitril–valsartan fluctuated from 25.2 to 180.7 USD/month, the ICER ranged from 1762.5 to 13,462.8 USD/QALY, still lower than three times the *per capita* GDP in China in 2021. Other factors had little impact on the ICER fluctuation.

### Probabilistic sensitivity analysis

**FIGURE 4:**
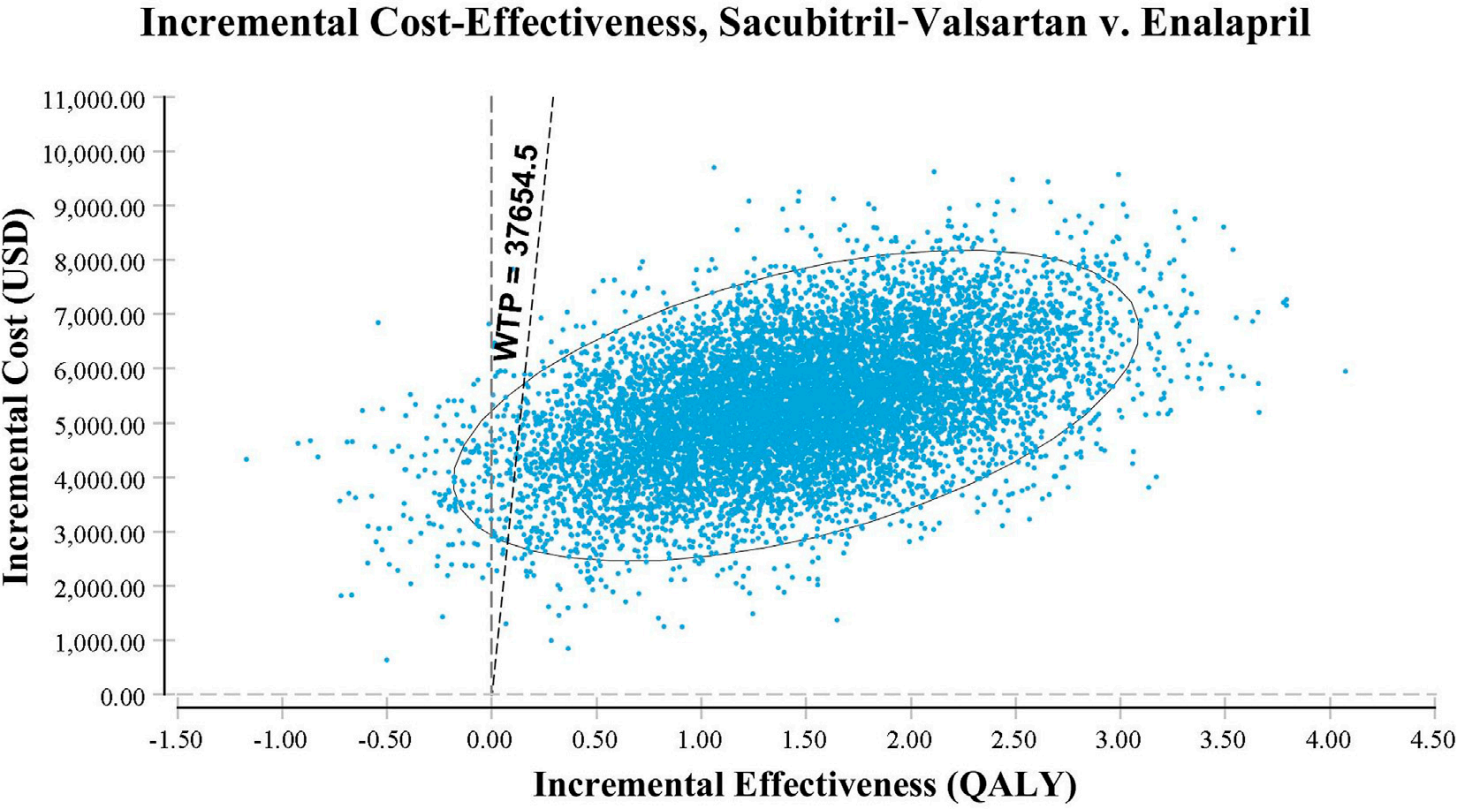
Scatter plot based on probabilistic sensitivity analysis. The probability that sacubitril–valsartan is cost-effective or superior to enalapril is over 97%.

**FIGURE 5:**
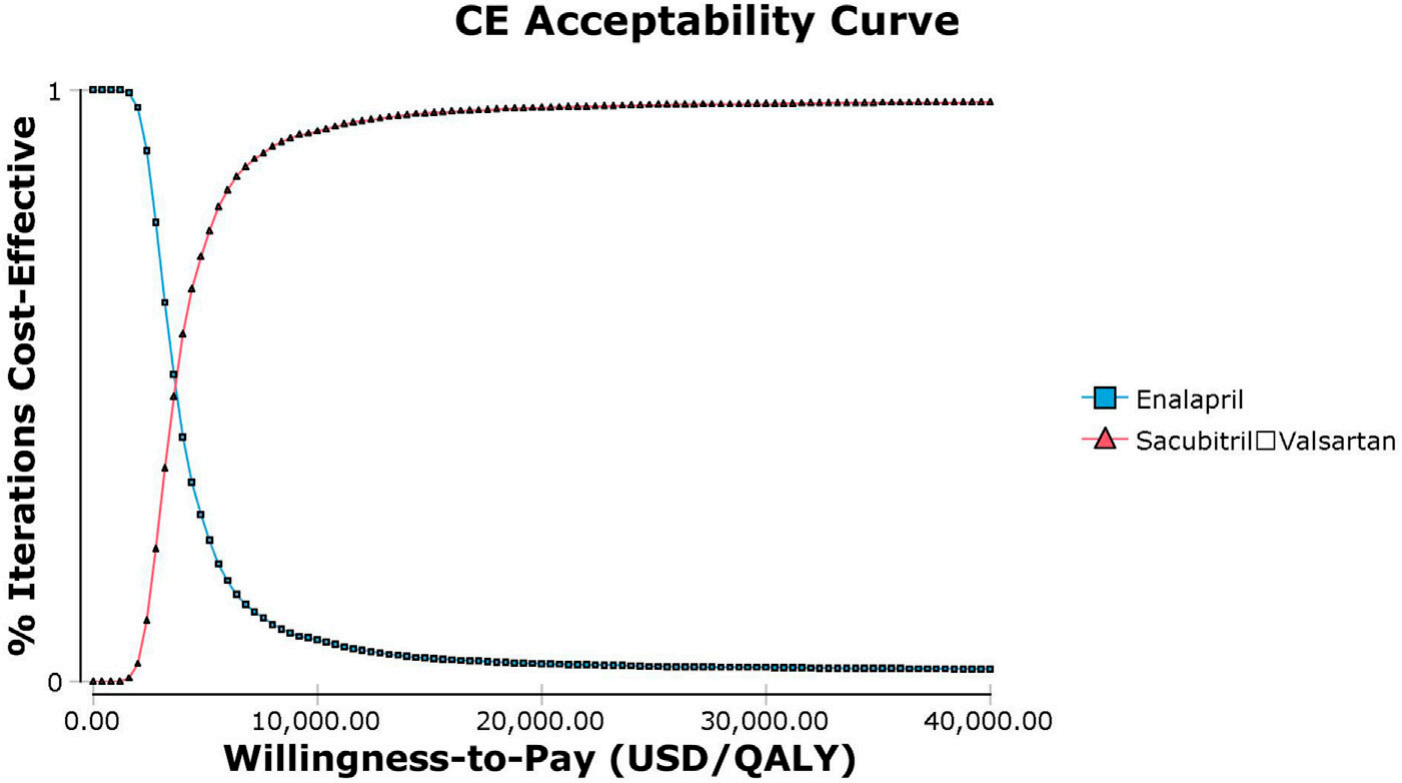
Abbreviation: CE, cost effectiveness. Cost-effectiveness acceptability curve of sacubitril–valsartan *versus* enalapril in acute decompensated heart failure in Chinese settings. When the willingness-to-pay threshold is 3,681.3 USD/QALY (0.293 times the *per capita* GDP in China in 2021), sacubitril–valsartan and enalapril shared the similar acceptability.

Probabilistic sensitivity analysis using Monte Carlo simulations based on probabilistic sensitivity sampling was conducted to validate the robustness of the results. In Figure 4, the scatter plot illustrated that under 97.4% of circumstances, sacubitril–valsartan was cost-effective or superior to enalapril when the WTP was 37,654.5 USD/QALY. Sacubitril–valsartan was not cost-effective or inferior to enalapril only in 2.6% of circumstances. The cost-effectiveness acceptability curve suggested that when the WTP was 3,681.3 USD (0.293 times the *per capita* GDP in China in 2021), sacubitril–valsartan and enalapril shared the similar acceptability, and when the WTP was higher than that value, sacubitril–valsartan gained higher acceptability than enalapril. When the WTP was 37,654.5 USD/QALY, the acceptability of sacubitril–valsartan was over 97% (Figure 5).

## Discussion

A previous economic evaluation of sacubitril–valsartan in Chinese settings has demonstrated that sacubitril–valsartan is cost-effective in stable HFrEF patients (Wu et al., 2020). Our study is the first to investigate sacubitril–valsartan in Chinese ADHF patients and found that early initiation of sacubitril–valsartan after stabilization of ADHF is cost-effective compared with enalapril; late initiation of sacubitril–valsartan after stabilization of HF is still cost-effective, even though not as cost-effective as early initiation of sacubitril–valsartan.

Sacubitril–valsartan is a combination of sacubitril and valsartan in equal proportions (Entresto, 2015). Sacubitril works by inhibiting neprilysin and enhancing the effect of natriuretic peptide, causing vasodilation, and the effects of diuretic and natriuretic peptides, ultimately reducing ventricular preload and remodeling (Mangiafico et al., 2013). Valsartan is a classical angiotensin Ⅱ receptor blocker that inhibits angiotensin II by blocking angiotensin Ⅱ receptor 1, causing vasodilation, and diuretic and natriuretic peptides, inhibiting aldosterone release (Wang et al., 2015; von Lueder et al., 2015). In addition to the abovementioned effects, sacubitril–valsartan could also function by improving endothelial dysfunction and arterial stiffness and by reducing oxidative stress, platelet activation, and inflammation circulating biomarkers (Cassano et al., 2022). Other drugs that could improve biomarkers of endothelial dysfunction and inflammation in hypertension might also improve the clinical prognosis of HF patients (Alshahawey et al., 2017; Ateya and Sabri, 2017; Alshahawey et al., 2019).

Sacubitril–valsartan has been proven effective in HFrEF patients in a large randomized controlled trial (RCT) (McMurray et al., 2014; Pascual-Figal et al., 2021). Wu’s study suggested that sacubitril–valsartan was cost-effective for Chinese HFrEF patients from the patient’s perspective (Wu et al., 2020), which may partly be due to the drug collective purchase policy and reimbursement policy. In 2017, the Chinese government launched the drug collective purchase policy to improve the healthcare quality (China National Healthcare Security Administration, 2023). Drugs only with cost-effectiveness could be included in the collective purchase lists, and drugs in the lists could be widely used in Chinese public hospitals, which provide over 80% of healthcare in China. The costs of sacubitril–valsartan (200 mg/tablet) have decreased from 19.43 CNY (3 USD) to 5.43 CNY (0.84 USD) since it was included in the list. On the other hand, the 80% reimbursement policy in Wu’s study also contributed to the cost-effectiveness of sacubitril–valsartan (Wu et al., 2020). Although sacubitril–valsartan was cost-effective for HFrEF patients from the patients’ perspective, we had no knowledge whether it was still cost-effective from the healthcare provider’s perspective, without consideration of any reimbursement policy. In addition, we also did not know whether sacubitril–valsartan should be used in ADHF patients as early as we can. In our study, there are two comparators; one had an early initiation of sacubitril–valsartan in the ADHF hospitalization period, and the other had an initiation of sacubitril–valsartan after the stabilization from HF, defined as not hospitalized for at least three consecutive months. Our results indicate that early initiation of sacubitril–valsartan can additionally gain 1.45 QALY and 1.61 life years, and the ICER is 23,628 CNY (3,662.4 USD)/QALY, far lower than the WTP of 37,654.5 USD. In addition, even though not equal to early initiation of sacubitril–valsartan, the initiation of sacubitril–valsartan after the stabilization from the HF event still could gain more benefit with less costs; the late initiation of sacubitril–valsartan gains 0.97 QALY, and the ICER is 28,673 CNY (4,444.4 USD)/QALY, still far lower than the WTP. Our results indicate that early initiation of sacubitril–valsartan is most cost-effective, and the late initiation of sacubitril–valsartan is still cost-effective. Chinese ADHF patients should initiate the sacubitril–valsartan treatment early in their hospitalization period to get better clinical outcomes and higher cost-effectiveness.

From the point of view of cost-effectiveness, the early initiation of sacubitril–valsartan remains controversial. A study conducted in the US showed that initiation of sacubitril–valsartan during hospitalization could reduce hospitalization incidence, increase quality-adjusted life years, and was cost saving compared with no initiation or initiation after HF stabilization (Gaziano et al., 2016). Another study conducted in Thailand confirmed this conclusion in their settings (Krittayaphong and Permsuwan, 2021). However, the study performed in Australia revealed that the current acquisition price could not make sacubitril–valsartan cost-effective (Perera et al., 2021). The WTP thresholds in China were lower than those in the US and Australia (Gaziano et al., 2016; Chin et al., 2020; Perera et al., 2021), but we still found that early initiation of sacubitril–valsartan was cost-effective, which may partly be attributed to the lower costs of sacubitril–valsartan in China. As mentioned previously, the collective purchase policy has made the drug costs decrease from 19.43 CNY (3 USD) to 5.43 for each tablet (equal to 0.84 USD), lower than that in the US and Australia, even lower than that in Thailand (Krittayaphong and Permsuwan, 2018). Another reason may be that the absolute value is great in reducing the events’ incidence (Packer et al., 2015). For a 30-day HF readmission, the incidence is 13.4% for enalapril but 9.7% for sacubitril–valsartan, which results in an absolute reduction of 3.7% (Desai et al., 2016). The reduction of cardiovascular mortality is still significant, and the cardiovascular mortality rate in the 2-month follow-up period after ADHF hospitalization is 3.4% and 2.3%, respectively. In our simulation, early initiation of sacubitril–valsartan could lead to an additional 1.45 QALY (or 1.61 life years). Even late initiation of sacubitril–valsartan still gained 0.97 QALY compared with the use of enalapril. The benefit in QALY and life years is almost consistent with that in Gaziano et al. (2016). To validate the robustness of our study, sensitivity analysis was performed; when the higher range of sacubitril–valsartan of 1,165.7 CNY (180.7 USD)/month was employed, which was the price of sacubitril–valsartan before including in the collective purchase lists, the ICER obtained was still lower than the WTP. Other factors had little impact on the ICER fluctuation. The probabilistic sensitivity analysis also showed that under 97.4% of circumstances, sacubitril–valsartan is cost-effective, indicating that our results are robust.

To improve healthcare quality, many programs have been established in China. In the China Heart Failure (China-HF) Registry launched in 2012, the in-hospital mortality was 4.1 ± 0.3% (Zhang et al., 2017), but it decreased to 2.8% in the latest Heart Failure Report in China (Working Group on Heart Failure NCfCQIN, 2021). We noticed that in 2017, sacubitril–valsartan accounted for about 2.3% of the overall oral RAAS inhibitors, but in 2020, it had risen to 63.7%, partly due to the acceptable costs. The wide use of sacubitril–valsartan has improved clinical outcomes to some extent, along with the use of other novel drugs. In addition, the indications for the treatment of hypertension with sacubitril–valsartan have been proven by China’s National Medical Products Administration, which may further improve the quality of Chinese HF patients.

In addition to sacubitril–valsartan, vericiguat and sodium-dependent glucose transporters 2 inhibitors (SGLT2i) have also been proven effective in ADHF or acute HF treatment (Armstrong et al., 2020; Bhatt et al., 2021). In the VICTORIA study, researchers investigated the efficacy of vericiguat on patients who had worsening HF and found that the incidence of cardiovascular death or hospitalization for HF was lower among those who received vericiguat than among those who received a placebo (Armstrong et al., 2020). The EMPULSE study and SOLOIST-WHF study demonstrated that initiation of SGLT2i in patients who had worsening HF or were hospitalized for acute HF could result in significant clinical benefits. Currently, vericiguat and several SGLT2i agents have been approved to treat HF in China, and SGLT2i and sacubitril–valsartan have been included in the collective purchasing list. The use of SGLT2i and sacubitril–valsartan in Chinese HF patients has climbed in the past few years. The real-world study of vericiguat, SGLT2i, and sacubitril–valsartan is warranted.

There are some limitations to our study. First, the data in our study were derived from large RCTs, which may not completely represent the patients in China, but a study investigated the efficacy of sacubitril–valsartan in HF and found that the differences in races did not modify the benefit of sacubitril–valsartan (Kristensen et al., 2016). Second, the costs in our study were derived from China’s local data, and whether this conclusion could be extended to other regions remains unclear. Third, we only used direct medical costs and direct non-medical costs, and indirect costs were not included in our costs; this limited us to analyzing it from the society’s perspective, which is the most comprehensive perspective. Fourth, the transition probability of clinical outcomes was derived from published studies rather than from the raw data, which limited us to perform subgroup analysis. Lastly, this study was conducted using a mathematical model. The costs and effectiveness of the model were obtained from published studies, and research based on real-world data is needed to confirm our conclusion.

## Conclusion

Early initiation of sacubitril–valsartan after stabilization of ADHF is of high cost-effectiveness compared with the use of enalapril. Late initiation of sacubitril–valsartan after stabilization of HF is still cost-effective but not as cost-effective as the early initiation of sacubitril–valsartan in ADHF. For Chinese ADHF patients, the time to initiate sacubitril–valsartan should be when the patient is stabilized from ADHF rather than stabilized from HF, from the perspective of economic evaluation.

## Data Availability

The original contributions presented in the study are included in the article/supplementary material; further inquiries can be directed to the corresponding author.
